# A decade of user operation on the macromolecular crystallography MAD beamline ID14-4 at the ESRF

**DOI:** 10.1107/S0909049509035377

**Published:** 2009-10-07

**Authors:** Andrew A. McCarthy, Sandor Brockhauser, Didier Nurizzo, Pascal Theveneau, Trevor Mairs, Darren Spruce, Matias Guijarro, Marc Lesourd, Raimond B. G. Ravelli, Sean McSweeney

**Affiliations:** a European Molecular Biology Laboratory, 6 rue Jules Horowitz, BP 181, 38042 Grenoble, France; b Unit of Virus Host Cell Interactions, UJF-EMBL-CNRS, UMI 3265, 6 rue Jules Horowitz, 38042 Grenoble Cedex 9, France; c European Synchrotron Radiation Facility, 6 rue Jules Horowitz, BP 220, 38042 Grenoble, France

**Keywords:** macromolecular crystallography, X-ray beam quality, beamline diagnostics, beamline automation

## Abstract

ID14-4 at the ESRF is the first tunable undulator-based macromolecular crystallography beamline that can celebrate a decade of user service. During this time ID14-4 has not only been instrumental in the determination of the structures of biologically important molecules but has also contributed significantly to the development of various instruments, novel data collection schemes and pioneering radiation damage studies on biological samples. Here, the evolution of ID14-4 over the last decade is presented, and some of the major improvements that were carried out in order to maintain its status as one of the most productive macromolecular crystallography beamlines are highlighted. The experimental hutch has been upgraded to accommodate a high-precision diffractometer, a sample changer and a large CCD detector. More recently, the optical hutch has been refurbished in order to improve the X-ray beam quality on ID14-4 and to incorporate the most modern and robust optical elements used at other ESRF beamlines. These new optical elements will be described and their effect on beam stability discussed. These studies may be useful in the design, construction and maintenance of future X-ray beamlines for macromolecular crystallography and indeed other applications, such as those planned for the ESRF upgrade.

## Abstract 2

The improvement of the X-ray beam quality achieved on ID14-4 by the installation of new X-ray optical elements is described.

## 1. Introduction

**Figure 1:**
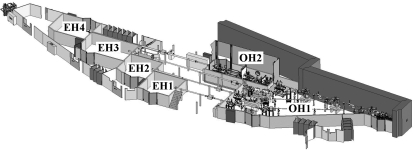
Schematic view of the ID14 beamline showing the optical and experimental hutches.

ID14-4 was commissioned in 1998 and has been instrumental in the determination of the structures of many biologically important structures, including the 30S ribosomal subunit (Wimberly *et al.*, 2000 ▶), tubulin (Ravelli *et al.*, 2004 ▶) and the complement component C3 (Janssen *et al.*, 2005 ▶), as well as making a significant contribution to structural studies on the 70S ribosome (Selmer *et al.*, 2006 ▶). ID14 is situated on an ESRF high-β straight section with three undulators in tandem and contains four end-stations, one tunable and three fixed-energy (Wakatsuki *et al.*, 1998 ▶) (Fig. 1 ▶). ID14-4 was commissioned with a fixed-exit Kohzu double-crystal monochromator (DCM) for energy selection and a toroidal mirror to focus the X-ray beam. However, later experience gained from the operation of other macromolecular crystallography (MX) beamlines at the ESRF has shown that the channel-cut monochromators of both ID23-1 (Nurizzo *et al.*, 2006 ▶) and ID29 are more reliable, easier to maintain and less sensitive to parasitic vibrational effects than a DCM.

In order to benefit from these advantages and to simplify maintenance across all ESRF MX beamlines the original DCM has been exchanged for a standardized ESRF channel-cut monochromator. The toroidal focusing mirror must be displaced in order to track beam movements resulting from tuning of the channel-cut monochromator. The innovative approach employed on ID23-1 (Nurizzo *et al.*, 2006 ▶) has proven to be reliable and conducive to automated beamline alignment. It was therefore decided to replace the original mirror and vessel with a set-up similar to ID23-1. This upgrade of the optical components complements an earlier refurbishment of the experimental hutch. These improvements, when combined with the continued improvement of automation protocols (Beteva *et al.*, 2006 ▶), now allow standard X-ray diffraction experiments, including multiple anomalous diffraction (MAD), to be easily and robustly performed. The investment in new equipment has also allowed the development of standardized beamline control software that can now be remotely operated (Gabadinho *et al.*, 2008 ▶; Soltis *et al.*, 2008 ▶).

ID14-4 was the first tunable undulator-based beamline available at the ESRF wholly dedicated for MX. Radiation damage was observed as a particular problem and an active collaboration between users and beamline scientists has significantly contributed to the scientific understanding of radiation damage in biological samples (Colletier *et al.*, 2008 ▶; Fütterer *et al.*, 2008 ▶; Owen *et al.*, 2006 ▶; Ravelli & McSweeney, 2000 ▶). Owing to its close association with the EMBL-Grenoble outstation, ID14-4 has also contributed to the development of many scientific instruments, such as the microspectro­photometer (McGeehan *et al.*, 2009 ▶) and the mini-κ goniometer head (MK3). In addition, ID14-4 has contributed to the development of novel methods for macromolecular crystallography, such as *de novo* phase determination using radiation damage (Nanao *et al.*, 2005 ▶) and X-ray tomographic reconstructions of macromolecular samples (Brockhauser *et al.*, 2008 ▶). Because of technological developments, beamlines at synchrotrons are continually evolving and ID14-4 is such an example. Here, we describe in detail a decade of systematic improvements on ID14-4 that will be beneficial to the construction of other synchrotron radiation beamlines.

## 2. Original beamline layout and optics

**Figure 2:**
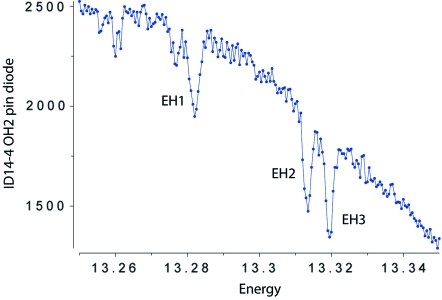
Energy scan of the ID14 diamonds using the pin diode directly after the monochromator showing the glitches for each of the three (EH1, EH2 and EH3) diamonds.

ID14 was designed so that four beamlines could operate simultaneously from one straight section (Fig. 1 ▶) by using three undulators (42, 24 and 23 mm periods). The three fixed-energy stations make use of the high intensity provided by the 23 mm-period undulator at 13.3 keV and use thin asymmetrically cut diamond crystals for energy selection. The other two undulators are used for experiments on ID14-4. The X-ray beam used for ID14-4 passes through the diamond monochromators, each selecting a portion of the ID14 X-ray beam around 13.3 keV, and producing a major glitch (∼20% of intensity) of width 10 eV at the beamline operating energy (Fig. 2 ▶); other minor glitches are also observed. The diamonds and all the optical elements for ID14-1, 2 and 3 are housed in one large optical hutch (OH1). Also housed in OH1 were three white-beam attenuators, four water-cooled blades software coupled in horizontal and vertical pairs as primary slits for beam size definition, and a similar four-bladed set-up of secondary slits to clean the X-ray scattering from the primary ones.

All the optical elements for ID14-4 are housed in a separate optical hutch, OH2, allowing the other beamlines to operate normally during maintenance on ID14-4 (Fig. 1 ▶). The ID14-OH2 hutch, when originally commissioned, contained four water-cooled blades software coupled in horizontal and vertical pairs as beam definition slits at its entry and three white-beam attenuators, allowing the heat load on the monochromator to be varied. Monochromatic X-rays were produced using an EMG-T5 Kohzu DCM manufactured under contract for the ESRF, with the rotation axis driven by a McLennan controller (Ash Vale, UK). An X-ray beam position monitor (XBPM) based on a four-pin diode and a retractable fluorescence screen were installed just after the monochromator for an intensity feedback loop, beam diagnosis and alignment procedures. A toroidal mirror, situated 20.5 m from the sample position, was used to focus the beam (demagnification ratio 2.3). Lastly, there was again a similar four-bladed set-up of horizontal and vertical slits to try to minimize any major beam drifts in the experimental hutch.

### 2.1. Double-crystal monochromator

The EMG-T5 Kohzu DCM has the rotational axis coupled to a translational cam assembly system, thus ensuring a fixed exit for the X-ray beam. The first crystal was predicted to receive a heat load of 120 W and was therefore liquid-nitrogen cryo-cooled, with the second crystal cooled *via* braided link. Initial instabilities were reduced with the installation of a Compton scattering shield for the second crystal and improved thermal isolation of the mechanical support from the monochromator crystal mounting. However, these measures were still insufficient. In addition, a Queensgate Instruments (Torquay, UK) controlled piezo feedback system was installed on the second crystal. Here, the intensity after the monochromator was monitored on the quadrant photodiode monitor and maximized by continuously adjusting a piezo actuator on the second crystal. This proved to be the single most important device that allowed ID14-4 to routinely operate. Later, cryo-cooling of the second crystal was tested and found to reduce the thermal beam drifts. An unfortunate side effect was some unwanted X-ray beam instabilities owing to the flexible cryo-pipes between the first and second crystals (Lesourd *et al.*, 2002 ▶).

**Figure 3:**
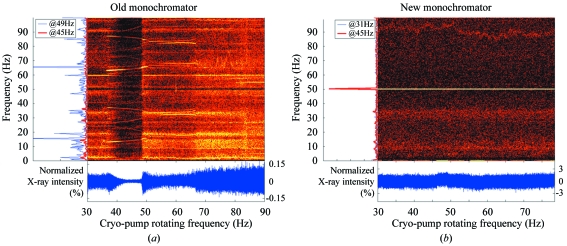
Time–frequency plots of the vibrational analysis on (*a*) the Kohzu monochromator and (*b*) the channel-cut monochromator. The horizontal axis represents time (proportional to the cryo-pump frequency) while the vertical axis is the frequency of the measured vibration signals.

Studies of the vibrational spectra of the X-ray beam revealed that the flexible cryo-pipes could resonate at certain cryo-pump speeds (Fig. 3*a* ▶). Furthermore, parasitic vibrations propagated through the experimental floor could cause the whole monochromator housing to resonate (Lesourd *et al.*, 2002 ▶). Many studies were undertaken to identify and subsequently remove or dampen the source of these parasitic vibrations, which originated from pumps, fans and water-cooling devices in the vicinity and not necessarily on ID14-4. Further reductions in some vibrational frequencies were obtained by installing vibrational dampers on the monochromator support. Despite these efforts, the piezo feedback system was still necessary to compensate for both residual short-term vibrations and persistent long-term (> minutes) drifts. Some newer laminar-flow flexible pipes with internal braids from Witzenmann (Thorigny sur Marne, France) were tested in 2005. These significantly reduced the vibrational effects on the X-ray beam but their increased rigidity hindered the alignment of the second crystal. Subsequently, the installation of stronger motors (Phytron VSH 65-200-5-UH) acting on the mechanical support of the second crystal and their use for alignments helped to overcome this problem but the energy scans were unacceptably slow.

### 2.2. Toroidal mirror

The original mirror on ID14-4 was fabricated in 1997 with a slope error of 6.8 µrad and significant non-uniformity, where local defects could reach 60 µrad. These defects were estimated to reduce the reflectivity at the focus position by an estimated 20–25% and to create a significantly non-uniform X-ray beam. The mirror was mounted in an ultra-high-vacuum vessel and bent using an air-pressure solenoid valve. The vessel was subsequently mounted on a metal frame. The mirror movements were controlled *via* five motors (three vertical and two horizontal) acting directly on the mirror through bellows. Small movements of the X-ray beam correlating to floor vibrations were also observed on the mirror, and suggested their transmission *via* the metal frame. However, the biggest problem turned out to be the erratic vertical movements of the mirror which made it difficult to reproducibly align. Originally, there was no X-ray beam visualization possible directly after the mirror to monitor this. External encoders were therefore installed to provide a long-term verification of the mirror motor positions and a beam viewing device was later installed just after the mirror. From these devices it was evident that while the motors moved correctly the mirror often did not move to the expected position and that there was an inherent mechanical problem with the coupling between the mirror and the three legs acting on it. Fortunately, the cumbersome alignment of the mirror was usually only carried out during start-ups since optical interventions were not frequently necessary owing to the fixed-exit DCM. It should also be noted that from time to time we experienced problems with the compressed-air bending mechanism. Although these were minor and mainly involved a repair of the solenoid valve, they could persist for weeks, as their effect could remain unnoticeable for the users and most local contacts. More rigorous quality-control protocols, such as the systematic checking of historical databases logging beamline parameters, are now implemented to try to prevent similar occurrences.

## 3. New beamline layout and optical elements

A number of modifications have been made to ID14-OH1 that are relevant to ID14-4. Firstly, the three white-beam attenuators were refitted with new pyrocarbon and Al foils. In order not to melt the Al foils in the white beam we have implemented a software block on the movement of the Al foils unless a pyrocarbon foil has first been inserted. The second modification was the installation of white-beam diagnostic elements (Mairs *et al.*, 2005 ▶) after the entry slits and both the EH1 and EH2 diamonds. These diagnostic elements can be inserted into the white beam and are composed of a motorized water-cooled copper block containing both a mechanically clamped chemical-vapour-deposited (CVD) diamond and a pyrocarbon foil at 45° to the beam. The CVD diamond fluoresces with visible light when illuminated with X-rays. This light is captured by a black-and-white video camera connected to either a screen or a four-channel Axis Communication (Lund, Sweden) A240Q video encoder box that can be used as a web-based video server for display on a PC in the control cabin. When the pyrocarbon foil is inserted into the beam a silicon diode is used to record its scattering through a Novolec amplifier that is read *via* WAGO devices (Table 1 ▶). The last and most recent development has been the replacement of the original four-bladed primary slits after the front end with new high-powered Cu-block-based primary slits (Marion & Zhang, 2003 ▶) in preparation for a possible increase in the ring current to 300 mA. These are similar to those installed on ID23-1 (Nurizzo *et al.*, 2006 ▶) and ID29 with a simple procedure for initialization, calibration and alignment.

**Figure 4:**
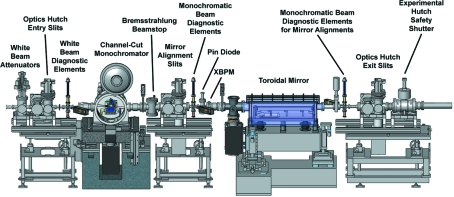
Schematic showing the layout of the optical elements of ID14-OH2.

From the beginning it was decided to keep the original ID14-OH2 shielding in an effort to minimize the beamline down time, budget costs and any disturbance to the operational fixed-energy beamlines. The large distance between the mirror and sample position is due to the original space constraints and the quality of focusing mirrors that could be fabricated in the mid-1990s. The ID14-4 user community also expressed their wish to keep as large a beam as possible on ID14-4. Such an option allows users the choice of adjusting the beam size to optimally match the crystal size for maximizing the signal-to-noise ratio. Therefore the optical hutch was rebuilt using a similar layout to the original one, except for the inclusion of slits after the monochromator and the incorporation of some additional X-ray beam diagnostic elements (Fig. 4 ▶).

Earlier vibrational studies had identified many parasitic vibrations as causes of unwanted movements on the X-ray beam position and intensity. It was therefore decided to minimize these effects by mounting all the optical elements on granite blocks that sit on top of concrete bases, thereby reducing any residual vibrations. To further reduce vibrational noise all the rotary pumps were moved outside the optical hutch and placed on vibrational damping pads. From outside they were connected to their respective turbo pumps *via* feed-through pipes passing through chicanes and valves. The three original white-beam attenuators were kept, refitted and controlled similar to those in OH1. An additional white-beam diagnostic element, as described above, was also installed in OH2 just after the ID14-OH2 entry slits for checking the diamonds and aligning the slits.

### 3.1. Monochromator system

**Figure 5:**
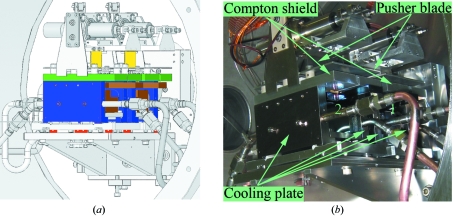
(*a*) Technical drawing of the channel-cut crystal mounted on ID14-4. The crystals are coloured in brown, the cooling plates in blue, the Compton scattering shield in yellow, the pusher blade in yellow and the ceramic thermal isolation elements in red. (*b*) The monolithic Si[111] crystals as currently mounted in the ID14-4 monochromator.

The new monochromator on ID14-4 is a vertical offset liquid-nitrogen-cooled channel-cut monochromator. The vessel is large enough to accommodate two monolithic Si crystals (Fig. 5 ▶) and either can be used by simply translating the whole vessel horizontally. At present there are two Si[111] crystals installed and it takes approximately one hour to change between them and realign the beamline. A standardized channel-cut monochromator vessel and crystal support was designed at the ESRF and manufactured under contract by Ateliers-Peyronnard (Champ sur Drac, France). The high-quality Si[111] crystals were cut and polished at the ESRF. The centre of rotation of the first crystal was aligned with respect to the X-ray beam path with the help of the ESRF Alignment Group by using the external motors that move the whole vessel vertically and horizontally. The crystal design and cooling scheme is an evolution of that used on ID23-1, ID29 and other beamlines at the ESRF. The channel-cut Si[111] crystals contain a ‘weak link’ between the larger diffracting face and the smaller second diffracting face. In order to adjust the pitch of the second diffracting surface a thin metal blade is used to deform the weak link integrated in the monolithic silicon crystal (Fig. 5 ▶). This allows scanning of the rocking curves and the rejection of higher harmonics. The crystals are side-cooled using liquid nitrogen by means of copper exchanger plates. The plates are attached to the sides of the crystal using invar bolts that traverse the silicon. A 0.1 mm indium foil is inserted between the copper and silicon to improve thermal contact. A Compton scattering shield was also added to reduce any undesired thermal drifts. It is also possible to monitor the temperature of the crystals and their support *via* thermocouples that are read through WAGO devices (Table 1 ▶). Both crystals are similar in quality, allowing us to easily replace either for testing other channel-cut designs or Si diffraction planes, such as the Si[311]. We are currently using the outer (furthest from the feed-through axis) Si[111] crystal because it gives a slightly higher flux (∼15%) than the inner crystal. The origin of this difference is not known as the rocking curves for the two crystals are identical*.*

The Bragg angle is driven with a stepper motor through a recently installed IcePAP control electronic system that was developed at the ESRF (Table 1 ▶). This system has an active feedback on a high-resolution RON886 incremental encoder coupled to an IBV650 interface (Heidenhain, Traunreut, Germany). This set-up allows a 0.05 mdeg minimum step size, corresponding to around 0.1 eV at 12.67 keV. The encoder for the monochromator is rigidly connected to a differentially pumped feed-through shaft and thus directly measures the Bragg angle. The main disadvantage of a channel-cut monochromator is of course the intrinsic vertical movement of the beam when changing energy. However, this can be readily compensated for by adjusting the mirror and/or the experimental table height. The beam movement is calculated using a simple formula [Δ*h* = 2*g*cos(θ_new_) − 2*g*cos(θ_old_)], where Δ*h* is the height change, *g* is the gap between the crystals (5 mm) and θ is the Bragg angle. This displacement is then applied to the mirror and experimental table. The experimental table is then scanned vertically to ensure that the optimum intensity is achieved. An additional absolute encoder (Baumer GM400) with a resolution of 21 mdeg is mounted directly on the monochromator stepper motor and is read through a WAGO device (Table 1 ▶). The zero position for this encoder was set to correspond to an energy of 11.56 keV (near the Pt *L*_III_ absorption edge) during the commissioning. This external encoder allows the monochromator Bragg angle to be verified and can also be used as a reference during monochromator interventions or if the IcePAP controller fails. In such a situation the monochromator angle can be driven close to the reference energy and reset. The monochromator is then recalibrated as described below.

A Bremsstrahlung beamstop was inserted directly after the monochromator. It is composed of 80 mm-thick tungsten that contains a 4 mm × 4 mm hole for the monochromatic beam. The position of the hole can only be adjusted manually as a safety precaution and this was done during the commissioning stages with the help of the ESRF Alignment Group. Four blades that are coupled in pairs by software act as horizontal and vertical slits directly after the beamstop. This set-up is similar to ID23-1 and ID29 and facilitates the alignment of toroidal mirrors. An actuator holding a beam intensity monitor, a series of metal foils and monochromatic beam viewer was installed after these slits (Mairs *et al.*, 2005 ▶). The beam intensity monitor is composed of a kapton foil mounted at 45° to the X-ray beam and an associated pin diode (Hamamatsu). The diode is then read through a Novolec amplifier and a WAGO device (Table 1 ▶). It is also possible to leave the diode in the X-ray beam to monitor the monochromatic beam intensity during user experiments. The metal foils are inserted into the X-ray beam in order to record an XANES (X-ray absorption near-edge structure) spectrum around their respective absorption edges (iron *K*-edge 7.111 keV; copper *K*-edge 8.980 keV; platinum *L*_III_, *L*_II_ and *L*_I_ 11.563 keV, 13.272 keV and 13.881 keV, respectively). The most useful metal foil for ID14-4 is platinum with an *L*_III_ absorption edge which is close to the external encoder reference position of the monochromator. The XANES spectrum is recorded by inserting a retractable pneumatic activated pin diode just after the metal foil, which is read through a Novolec amplifier and a WAGO device (Table 1 ▶). An identical scan with the platinum foil removed is also performed to normalize for intensity fluctuations in the platinum foil absorption scan. A customized *Python* program developed on ID23-1 (Nurizzo *et al.*, 2006 ▶) then automatically calculates the inflection point and is used to calibrate the monochromator with a typical error of 0.3 eV. Just before the mirror there is an XBPM based on a four-pin diode that is read using a YMCS0012 current amplifier (FMB Oxford, Oxford, UK) and converted into an X-ray beam position and intensity by a linux-based device server.

### 3.2. Focusing mirror

**Figure 6:**
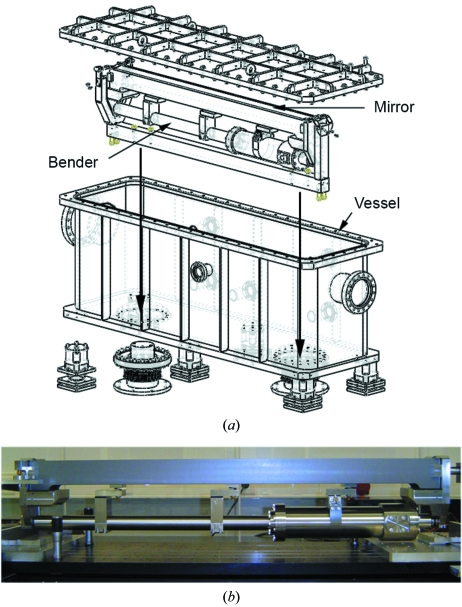
(*a*) Technical drawing of the toroidal mirror as mounted on ID14-4. The arrows indicate how the mirror is rigidly attached to the granite table. (*b*) A picture of the ID14-4 toroidal mirror taken in the metrology laboratory of the ESRF (courtesy of A. Rommeveaux, ESRF Metrology Group).

The new mirror mounting system is similar to that of ID23-1 (Nurizzo *et al.*, 2006 ▶). The mirror and vessel are independently fixed on a granite block as in Fig. 6 ▶ to ensure no detrimental vibrations are transmitted through the vessel to the mirror. The mirror can be aligned as a single unit using an ESRF designed motorized system from Cinel (Padova, Italy) that consists of five external motors acting on the granite block. The nominal angle of incidence of the mirror is 2.75 µrad. The large distance between the source and mirror means that the X-ray beam is very sensitive to any parasitic vibrational effects; however, testing of the new mirror mount system revealed a much improved vibrational stability, where a reduction from 2.8 to 1.3 in the amplification ratio for lateral vibrations transmitted from the floor to the mirror vessel could be achieved.

A state-of-the-art X-ray mirror and mechanical bending mechanism was fabricated and assembled by SESO (Aix-ex-Provence, France) to specifications that were validated by the ESRF Metrology Group. Tests on the unbent mirror showed that the longitudinal slope error was <1.2 µrad and that it had a surface roughness of 4.1 Å r.m.s.d. (root-mean-square deviation). The X-ray mirror is constructed from silicon and coated with palladium. The mirror is 900 mm long with a sagittal radius of 77.2 mm ± 1 mm and a nominal meridional radius of 9 km. The metrology results show that the radius of curvature repeatability at 9 km is better than 1.1% with a bending stability (Δ*R*c) of 18 m at 9 km.

During the initial installation of the mirror the ESRF Alignment Group ensured the precise positioning of the mirror in a flat orientation along the incident X-ray beam. The absolute external encoders mounted on the five mirror motors were then zeroed at this position and are now used as a reference in case of problems. To enable the automatic mirror alignment procedures developed at the ESRF we installed two HR50 Sony cameras cabled to a MATROX PCI card and controlled through a TACO device server. An actuator holding a diode and YAG screen was installed immediately after the mirror. The first camera is positioned to look at the YAG screen and is used for a pre-alignment procedure. This procedure is a slight modification to that described by Nurizzo *et al.* (2006 ▶) and ensures that the incident monochromatic beam hits the middle of an unbent mirror. In the first step, the camera region of interest (ROI) is set to continuously monitor the reflected beam on a slightly inclined mirror while it is vertically translated. By automatically capturing the mirror heights at the appearance and disappearance of the reflected beam the appropriate vertical height to centre the X-ray beam on the mirror can be determined. In the second step, the camera ROI is set to monitor a direct pencil-shaped beam that is typically defined as 0.1 mm × 0.1 mm by the slits before the mirror. The mirror is then scanned vertically at different horizontal positions. The correct horizontal position is calculated as the minimum of a regression parabola fitted on the different vertical heights. Although the mirror can be properly aligned using these measurements, the final focusing adjustment must be done at the sample position.

To complete the mirror alignment an X-ray scintillator based on a P43 phosphor screen Gd_2_O_2_S:Tb is mounted on a Sony HR50 camera. This camera is fixed to the X-ray diffraction detector and is driven close to the sample position. The *BeamFocus* software developed at the ESRF (R. Pieritz, personal communication, and Table 1 ▶) is then used to optimize the incident angle, yaw and longitudinal radius for the smallest possible beam displacements and corresponding to the smallest possible beam size. We are currently able to obtain a FWHM beam size of 90 µm vertically and 280 µm horizontally. This is much better than that previously achievable (250 µm and 320 µm) and is very close to the theoretically predicted size. This can be attributed to a much better mirror and more accurate control over the mirror’s position in space. These procedures allow the beamline staff to check the optimal focusing of the mirror during each machine restart (typically five times per year). This software can also be used to focus the X-ray beam on the detector if required for more complex experiments. Refocusing on the detector is not recommended on ID14-4 as the beamline is located on a high-β section with a very low divergence source (∼10 µrad horizontally and ∼3 µrad vertically). However, the mirror has often been unbent to increase the vertical size of the beam for the complete illumination of larger crystals.

## 4. User environment

**Figure 7:**
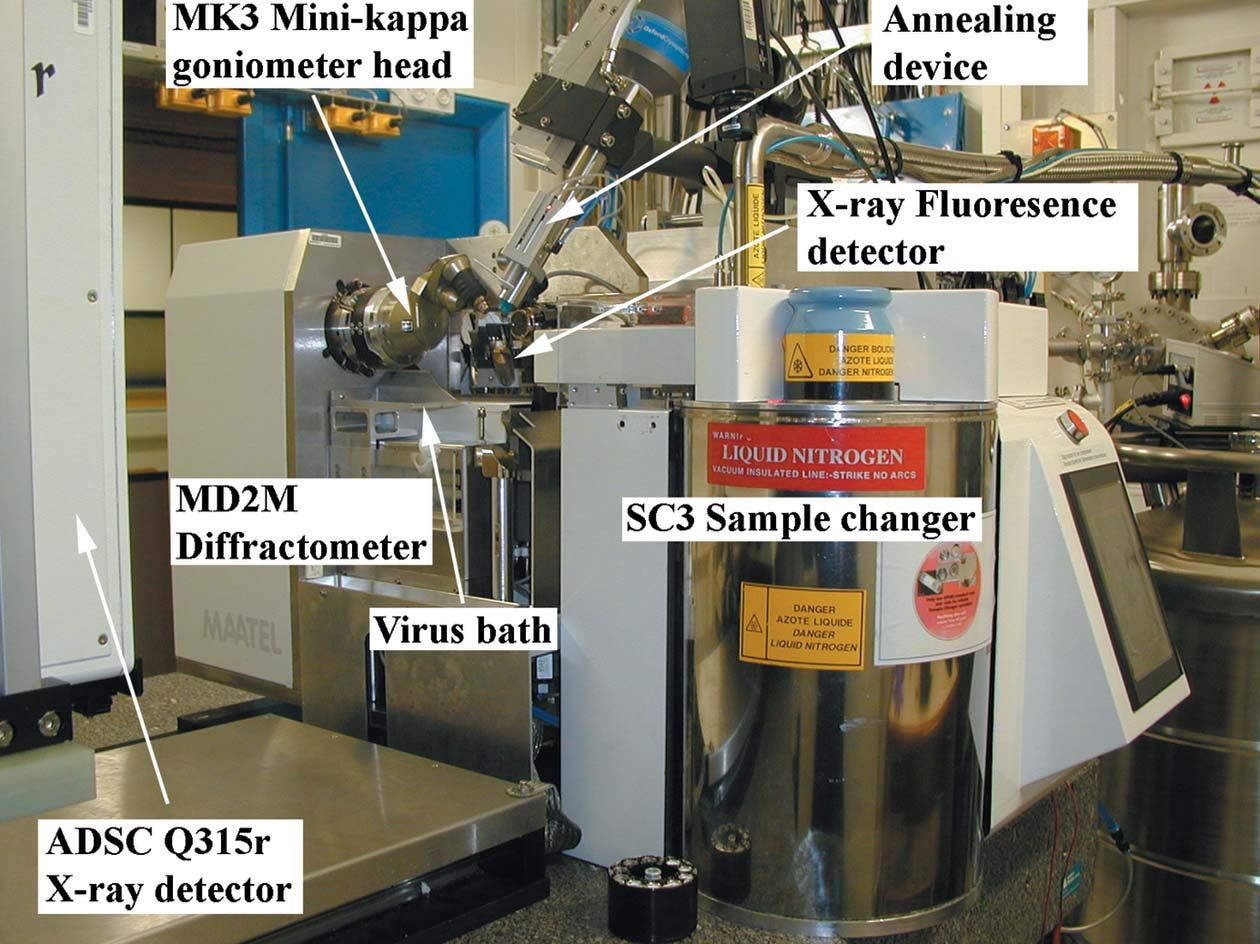
Photograph of the routine sample environment set-up in the ID14-4 experimental hutch.

**Figure 8:**
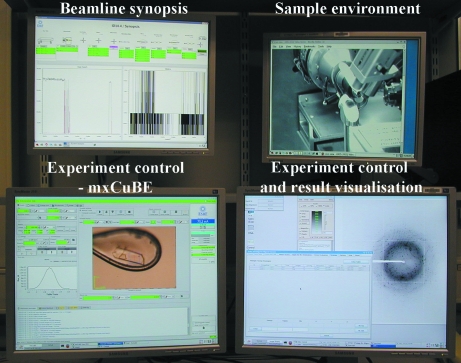
Photograph of the screen layout in the control cabin of ID14-4 showing the linux control PC dual monitors as well as two others for beamline diagnostics and web streaming displays.

The ID14-4 experimental end-station was extensively refurbished between 2004 and 2005 (Fig. 7 ▶). The only remaining piece of the original equipment is the experimental table on which all of the ancillary equipment is mounted. The control cabin is situated upstairs, as in the original layout. The control computer is a dual screen linux-based system with a number of desktops running various applications either locally or remotely (Fig. 8 ▶). Above the control computer are two additional screens, one for synoptic purposes and the other for video monitoring (Fig. 8 ▶). Here a number of cameras in the optical and experimental hutch are connected to two four-channel Axis Communications (Lund, Sweden) A240Q video encoder boxes and viewed as a web service. The cameras of the first encoder box are connected to beam viewers, and are routinely used by beamline staff to diagnose problems. The second encoder box is connected to cameras in the experimental hutch enabling both local and remote users to visually follow as the samples are loaded or unloaded by the sample changer and ready for centring.

### 4.1. Experimental hutch equipment

The X-ray beam enters the hutch and first passes through a series of vacuum Al filters that can be inserted to provide variable X-ray attenuation. These are followed by a vacuum slit-box containing four (two vertical and two horizontal) high-precision beam-defining/cleaning slits (JJ-XRAY, Lyngby, Denmark). Immediately after the beam-definition slits there is a scattering foil diode which can be read through a Novolec amplifier and WAGO device (Table 1 ▶). This diode is used for beamline alignments and is also calibrated to produce photon flux values. After the slit box there is a fast and robust piezo millisecond shutter (CEDRAT, Meylan, France) with strain gauge followed by another scattering foil diode that is read as above. These diodes, the ϕ-encoder signal and the millisecond shutter control are all connected to a MUSST (multipurpose unit for synchronization, sequencing and triggering) electronic card designed by the ESRF (Table 1 ▶). This configuration allows for a simple diagnosis of shutter/ϕ synchronization problems, often associated with poor data quality on an undulator-based beamline (Flot *et al.*, 2006 ▶). We also use this configuration to provide continuous integrated intensity measurements during data collections.

The sample environment is composed of a high-precision MD2M diffractometer equipped with on-axis microscope to avoid parallax error (MAATEL, Voreppe, France) (Perrakis *et al.*, 1999 ▶) and a MK3 goniometer head for crystal realignment (Fig. 7 ▶). The sample position is kept at 100 K by a Cryo-stream 700 series (Oxford Cryosystems, Witney, UK) mounted on a pneumatic actuator. A remotely controlled cryoshutter is installed on the cryo-stream and can be remotely controlled to anneal a frozen crystal sample as needed (Giraud *et al.*, 2009 ▶). Samples, mounted on SPINE standard cryopins, can be either mounted manually or more commonly with a Grenoble sample changer (SC3) (Cipriani *et al.*, 2006 ▶). The current X-ray detector installed on ID14-4 is the large-surface high-quality ADSC Q315r mosaic CCD detector (ADSC, Poway, CA, USA) with a fast parallel readout time of ∼150 ms.

It is often necessary to collect data at the peak of the absorption edge for a successful experimental phasing experiment. As this peak can vary, it is advisable to perform an energy scan near to the absorption edge to determine its exact position. ID14-4 has a high-dynamic-range energy-dispersive Xflash 1000 X-ray fluorescence detector (Bruker AXS, Madison, WI, USA) for such measurements. The ROI for a particular element is programmed on the MultiMax signal processing unit of the Xflash detector through a serial line connection. This selected region is then read back using a TTL (transistor–transistor logic) signal by the MUSST card, which is also connected to the monochromator encoder. This set-up enables the fluorescence signal to be read during a continuous motion of the monochromator and allows for XANES scans in the 20–30 s timescale. All of the energy scans performed on the public tunable MX-beamlines (ID14-4, ID23-1 and ID29) at the ESRF now use this configuration.

The MD2M has also been upgraded since its installation and commissioning in July 2005. One improvement is the use of a modified beamstop and cleaning aperture. The beamstop of the MD2 (Perrakis *et al.*, 1999 ▶) is situated 10 mm from the sample position and this has been exchanged for a longer one that is now 15 mm from the sample position. The cleaning aperture just before the sample and the small size of the beamstop (0.4 mm) at a longer distance results in the routine measurement of diffraction spots down to 50 Å resolution. A ‘virus bath’ has also been added to retrieve any potentially hazardous biological samples dropped during the loading and unloading of samples by the SC3. The original sample backlight illumination has been upgraded to a more homogeneous and robust LCD lighting device with a front lighting option for continuous visualization during a data collection. Another improvement was the replacement of an analogue video camera in the MD2M with a new Prosilica GC655C gigabit camera. This camera is now plugged directly into an Intel gigabit card on the linux-based control computer. This allows the display of uncompressed images and results in a video quality and frame refresh rate that is far superior to what was previously achieved.

### 4.2. Control software

Most of the beamline stepper motors are controlled *via* the standard VPAP/DPAP electronics system used at the ESRF. There is, however, a migration occurring at the ESRF to the newly developed IcePAP system (Table 1 ▶). For example, the recently installed primary slits are now controlled using this system. These and other control electronics, such as the Galil motion card (Galil, Rocklin, CA, USA) used to control the MD2M spindle axis, are all embedded in a *SPEC* environment and driven through device server software (Table 1 ▶). Two separate *SPEC* sessions are used to control the optics hutch (OH2) and experimental hutch (EH4) motors. All the beamline diodes, with the exception of the XBPM, are connected to *SPEC* through WAGO modules (Table 1 ▶). Other essential beamline devices, such as the Xflash fluorescence detector, can be controlled in *SPEC* *via* *TACO/TANGO* device servers. The very top layer allows all of the beamline components to be controlled through easy and user-friendly *Python*-based graphical user interfaces (GUIs) developed at the ESRF. A simple MXControl panel GUI is used to control the optics and experimental hutch motors, insert filters, beam viewers and diodes and also to display the diode values for general diagnostic purposes. The more complicated MxCuBE GUI (J. Gabadinho, manuscript in preparation) has been developed to control the complete MX experiment, from mounting and centring samples through to data collection and analysis. This interface communicates to *SPEC* through a dedicated port and through *TACO/TANGO* device servers to other essential beamline components such as the SC3. In addition, it allows the implementation of simple buttons for automatic beamline procedures, such as aligning the beamline and carrying out automatic edge scans. This software is continually evolving and includes some new features such as the ability to analyse a full X-ray fluorescence (Leonard *et al.*, 2009 ▶) using the *PyMCA* software (Solé *et al.*, 2007 ▶) developed at the ESRF. This software may also be used for diagnostic purposes and was recently used to identify a faulty detector on ID14-4. Here, a much larger energy resolution (350 eV) than specified in the technical documents (<150 eV) was observed. A closer examination showed that this was due to a fault in the Peltier cooling of the silicon diode. The detector was replaced and the faulty unit was sent back for repair. In fact, MxCuBE is now mature and robust enough to routinely allow remote data collection experiments (Gabadinho *et al.*, 2008 ▶), similar to those at the SSRL (Soltis *et al.*, 2008 ▶).

In addition to the software for operating the beamline there are many database developments. Two particularly important databases for all of the ESRF MX beamlines are the beamline historical database (HDB) and the ISPyB user database (Beteva *et al.*, 2006 ▶). The beamline HDB allows motor positions, beam intensity values and both optical device temperatures and vacuum values to be polled and recorded at regular intervals. The database can be interrogated to identify the source of problems and may even aid in flagging potential problems that require regular maintenance interventions, such as the refurbishment of vacuum pumps. The user database ISpyB allows the users to plan, track and log their experiments. This database will become more important as many users are now remotely controlling the beamlines from their home laboratories.

## 5. Conclusions and future perspectives

In summary, the extensive efforts of the incremental refurbishment of ID14-4 have been necessary for the continued success of the beamline over the last decade. The MD2M goniometer and the SC3 sample changer in combination with associated software developments have dramatically changed the macromolecular crystallography experiment over the last decade. Users now routinely screen a number of crystals before selecting the best one or two to measure their diffraction experiment from, something that was impossible in 1998. This allows a much more efficient use of available beam time. Other developments are continuing and the MK3 mini-κ goniometer head is now being routinely used since its permanent installation.

The new optical elements, and in particular the channel-cut monochromator, have resulted in a superior X-ray beam quality. The improvements include a reduction in the amplification ratio of lateral vibrations transmitted from the floor to the monochromator vessel. Here a reduction from 2.5 on the DCM to 1.3 for the channel-cut monochromator was achieved. A reduction in the ratio of instability of the X-ray beam intensity to the mean intensity was also observed; these ranged from an estimated 10–15% for the DCM to 4.4% for the channel-cut monochromator. These measurements, taken together, imply an improvement in the stability of between four- and six-fold for the channel-cut monochromator. It should also be noted that the position of the X-ray beam for the measurements on the channel-cut monochromator were offset to maximize the instability. However, the most important improvement is the dramatic reduction of the many frequency components that are excited by the turbulent flow of the cryo-cooling in the two monochromators (Fig. 3 ▶).

**Figure 9:**
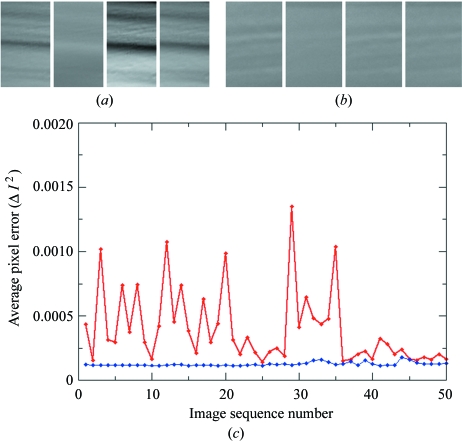
Low-frequency beam vibration study. Four subsequent dark- and flatfield-corrected images of the unfocused direct beam collected at the sample position (*a*) on the old set-up with the Kohzu monochromator and (*b*) on the new set-up with the channel-cut monochromator. (*c*) Plotting of the normalized intensity changes along the full 50 subsequent measurements on the old set-up is in red, while on the new set-up it is in blue.

These improvements are directly observed in both the X-ray crystallography data collection and X-ray tomography reconstruction experiments (Fig. 9 ▶). For example, we previously required three passes and exposure times of at least 1 s for optimal data collection statistics. Now we routinely use a single pass and exposure times of 100–200 ms, similar to ID23-1 and ID29, equating to a five- to ten-fold improvement. Other examples include the most recent X-ray tomographic reconstructions. Using the original optical elements required a more complex experiment and reconstruction algorithm than really necessary (Brockhauser *et al.*, 2008 ▶), whereas now, with a channel-cut monochromator, we can collect X-ray tomographic data in minutes and use much simpler and standardized reconstruction algorithms. The new optical elements have also reduced the maintenance work load for the EMBL and ESRF staff. Extensive efforts at improving the DCM often meant stressful interventions were necessary. Whereas the much more robust channel-cut monochromator is simpler to maintain and operate, the new monochromator also means that all the tunable MX beamlines now have fully compatible components and software. This allows the rapid deployment of newly developed software algorithms, such as fast energy scans, across the MX beamlines and the identification of potential problems at an earlier stage.

The continued success of ID14-4 over the last decade is due to the combined efforts of the EMBL and the ESRF in developing specialized beamline equipment and implementing new software algorithms. These efforts are continuing, and in March 2009 the original 42 mm-period undulator was replaced by a new 35 mm-period device. We hope the installation of this new undulator will minimize some of the current perturbations to the fixed-energy beamlines. This is now fully commissioned and ID14-4 has been completely refurbished from source to sample. All this major investment in new equipment should allow the transfer of operationally validated equipment to a new canted beamline (UPBL10) that is proposed to replace the existing ID14 in the ESRF upgrade (Detlefs, 2008 ▶; Morel, 2008 ▶).

Of course, the newer tunable beamline(s) proposed to replace ID14-4 will no doubt evolve to supersede ID14-4, which will probably cease to exist in 2012. However, much valuable information on beamline design and maintenance of an undulator MX beamline has been acquired from over a decade of user operation on ID14-4. It is difficult to predict how new X-ray beamlines will look like after the next decade, but some potential improvements include the focusing properties, a change in spindle orientation and new detector technologies. For example, the use of a KB mirror for a parallel microfocused beamline could be envisaged, perhaps even combined with a dual stripe toroidal mirror for a double-focusing possibility. One stripe would allow a medium (100 µm × 100 µm) beam size, while the second stripe would focus on a KB mirror close to the sample position for a microfocus beam size (<10 µm). In such a set-up the toroidal mirror can compensate for the change in beam height that occurs with channel-cut monochromator energy changes. Such a set-up also allows for the same sample environment to be used and a reduced intensity loss owing to size constraints on KB mirrors. Changing the ϕ-axis orientation could complement such a microfocus X-ray beam because preliminary studies on ID29 suggest that rotating about a vertically suspended spindle axis results in a much lower sphere of confusion (<0.5 µm), even when a MK3 mini-κ goniometer is used. This opens up the possibility of using the MK3 to orientate microcrystals, which is not recommended in the horizontal orientation configuration currently used on the microfocus beamline ID23-2 (Flot *et al.*, 2010 ▶). Further studies are required to determine how important the effect of the synchrotron polarization is on X-ray diffraction data and subsequent structural determinations. Of course, detector technology has changed considerably in the last few years and the whole MX community is excited by the arrival of the PILATUS (Dectris, Villigen, Switzerland) detectors. This detector technology opens up new and exciting possibilities, especially when combined with modern X-ray beamlines.

## Figures and Tables

**Table 1:** Summary of beamline ID14-4 parameters

X-ray source	23 mm-, 24 mm- and 35 mm-period undulators with minimum gap of 11 mm

Monochromator (Ateliers-Peyronnard, Grenoble, France)	
Crystal	2 × channel-cut double-crystal silicon [111]
Energy range	5.5–20 keV (owing to the absorption of the side-station diamonds the operational range is limited between 8.9 and 20 keV)
Rocking curve	30 µrad at 13.2 keV

Focusing element [toroidal mirror (Seso, France)]	
Surface coating	Pd
Useful area	620 mm × 5 mm
Sagittal radius	77.16 mm ± 1 mm
Meridional slope error	<1.2 µrad
Surface roughness	<4 Å
Focusing ratio	2.25 (46.2 m source to mirror, 20.5 m mirror to sample position)
Measured focus size at sample position	90 µm × 280 µm (V × H) FWHM
Measured flux at 13.2 keV with 200 mA current (photons s^−1^)	5.4 × 10^12^ (slit size 0.4 mm × 0.4 mm)
	4.1 × 10^12^ (slit size 0.2 mm × 0.2 mm)
	3.1 × 10^12^ (slit size 0.1 mm × 0.1 mm)

WAGO-I/O-System 750 (http://www.wago.com/)	
External encoders	750-630 (SSI transmitter interface)
Diode reading	750-467 (0–10 V) or 750-476 (±10 V)
Diode gain control	750-516 (digital output module)
Thermocouple reading	750-469 (analogue input module for thermocouples)
Pneumatic control	750-402 or 750-430 (digital input module)
Hardware permissions	750-513 or 750-517 (relay output module)

Relevant links	
ID14-4 web pages	http://www.esrf.eu/UsersAndScience/Experiments/MX/ID14-4/
	http://biosync.rcsb.org/esrf/ID14-4.html
Software	
*SPEC*	http://www.certif.com/
*TACO*/*TANGO*	http://www.tango-controls.org/
*BeamFocus*	http://beamfocus.sourceforge.net/
*PyMCA*	http://pymca.sourceforge.net/
*ISPyB*	http://www.esrf.eu/UsersAndScience/Experiments/MX/Software/ispyb

Electronics	
MUSST card	http://www.esrf.eu/UsersAndScience/Experiments/TBS/ISG/musst
IcePAP	http://www.esrf.fr/UsersAndScience/Experiments/TBS/ISG/icepap
